# Emerging SARS-CoV-2 mutation hot spots include a novel RNA-dependent-RNA polymerase variant

**DOI:** 10.1186/s12967-020-02344-6

**Published:** 2020-04-22

**Authors:** Maria Pachetti, Bruna Marini, Francesca Benedetti, Fabiola Giudici, Elisabetta Mauro, Paola Storici, Claudio Masciovecchio, Silvia Angeletti, Massimo Ciccozzi, Robert C. Gallo, Davide Zella, Rudy Ippodrino

**Affiliations:** 1 Elettra Sincrotrone Trieste - Area Science Park, Trieste, Italy; 2 Department of Physics, University of Trieste, Via Valerio 2, Trieste, Italy; 3 Department of Medicine, Surgery and Health Science, University of Trieste, Trieste, Italy; 4 Ulisse BioMed - Area Science Park, Trieste, Italy; 5 Institute of Human Virology, Department of Biochemistry and Molecular Biology, School of Medicine, University of Maryland, Baltimore, USA; 6 Medical Statistic and Molecular Epidemiology Unit, University of Biomedical Campus, Rome, Italy; 7 Institute of Human Virology, Department of Medicine, School of Medicine, University of Maryland, Baltimore, USA; 8 Co-founder and International Science Advisor - Global Virus Network, Baltimore, USA; 9 Member of the Global Virus Network, Baltimore, USA

**Keywords:** SARS-CoV-2, COVID-19, Mutation, Drug resistance, RdRp, Europe, RNA-dependent-RNA-polymerase, Pneumonia, Viral mutagenesis

## Abstract

**Background:**

SARS-CoV-2 is a RNA coronavirus responsible for the pandemic of the Severe Acute Respiratory Syndrome (COVID-19). RNA viruses are characterized by a high mutation rate, up to a million times higher than that of their hosts. Virus mutagenic capability depends upon several factors, including the fidelity of viral enzymes that replicate nucleic acids, as SARS-CoV-2 RNA dependent RNA polymerase (RdRp). Mutation rate drives viral evolution and genome variability, thereby enabling viruses to escape host immunity and to develop drug resistance.

**Methods:**

We analyzed 220 genomic sequences from the GISAID database derived from patients infected by SARS-CoV-2 worldwide from December 2019 to mid-March 2020. SARS-CoV-2 reference genome was obtained from the GenBank database. Genomes alignment was performed using *Clustal* Omega. Mann–Whitney and Fisher-Exact tests were used to assess statistical significance.

**Results:**

We characterized 8 novel recurrent mutations of SARS-CoV-2, located at positions 1397, 2891, 14408, 17746, 17857, 18060, 23403 and 28881. Mutations in 2891, 3036, 14408, 23403 and 28881 positions are predominantly observed in Europe, whereas those located at positions 17746, 17857 and 18060 are exclusively present in North America. We noticed for the first time a silent mutation in RdRp gene in England (UK) on February 9th, 2020 while a different mutation in RdRp changing its amino acid composition emerged on February 20th, 2020 in Italy (Lombardy). Viruses with RdRp mutation have a median of 3 point mutations [range: 2–5], otherwise they have a median of 1 mutation [range: 0–3] (p value < 0.001).

**Conclusions:**

These findings suggest that the virus is evolving and European, North American and Asian strains might coexist, each of them characterized by a different mutation pattern. The contribution of the mutated RdRp to this phenomenon needs to be investigated. To date, several drugs targeting RdRp enzymes are being employed for SARS-CoV-2 infection treatment. Some of them have a predicted binding moiety in a SARS-CoV-2 RdRp hydrophobic cleft, which is adjacent to the 14408 mutation we identified. Consequently, it is important to study and characterize SARS-CoV-2 RdRp mutation in order to assess possible drug-resistance viral phenotypes. It is also important to recognize whether the presence of some mutations might correlate with different SARS-CoV-2 mortality rates.

## Background

The recent emergence of the novel, human pathogen Severe Acute Respiratory Syndrome Coronavirus 2 (SARS-CoV-2) in China and its rapid national and international spread poses a global health emergency. On March 11th 2020, WHO publicly declared the SARS-CoV-2 outbreak as a pandemic. In a few weeks, the virus caused thousands of deaths worldwide, strongly impacting the global economy and human habits. SARS-CoV-2 is an enveloped, +ssRNA virus, belonging to the *Betacoronavirus* genus which includes two other RNA viruses that have caused recent important epidemics: Severe Acute Respiratory Syndrome (SARS) caused by SARS-CoV, and the Middle East Respiratory Syndrome (MERS) by MERS-CoV.

Noteworthy, some evidence has been recently provided, supporting that SARS-CoV-2 mortality can significantly differ depending on the geographic area. For example, Baud and colleagues reported that mortality rate is three times higher out of China (15.2% [95% CI 12.5–17.9] out of China, compared to 5.6% [95% CI 5.4–5.8] in China) [1]. This rate has been re-estimated by dividing the number of deaths on a given day by the number of patients with confirmed SARS-CoV-2 infection 14 days before, considering the WHO data relative to the cumulative number of deaths to March 1st, 2020 [1]. Differences in viral infection rates can be due to a combination of factors, including different national strategies adopted for people movement restrictions, isolation and quarantine, different genetic population herd immunity. Mortality differences are to understand, but viral mutations and evolution capability over time may be important.

RNA viruses mutation rate is dramatically high, up to a million times higher than that of their hosts and this high rate is correlated with virulence modulation and evolvability, traits considered beneficial for viral adaptation [2]. Wang and coworkers have recently characterized 13 variation sites in SARS-CoV-2 ORF1ab, S, ORF3a, ORF8 and N regions, among which positions 28144 in ORF8 and 8782 in ORF1a showed a mutation rate of 30.53% and 29.47%, respectively [3]. Prior reported results show that SARS-CoV-2 is rapidly moving across countries and genomes with new mutation hotspots are emerging.

RNA virus mutation rate contributes to viral adaptation creating a balance between the integrity of genetic information and genome variability [4–6]. Biological characterization of viral mutations can provide precious insights for assessing viral drug resistance, immune escape and pathogenesis related mechanisms. Additionally, viral mutation studies can be crucial for designing new vaccines, antiviral drugs and diagnostic assays. The viral genome mutagenic process depends on the viral enzymes that replicate the nucleic acids, influenced by few or no proofreading capability and/or post-replicative nucleic acid repair. Other mutation-generating processes include: host enzymes, spontaneous nucleic acid damages due to physical and chemical mutagens, recombination events and also particular genetic elements responsible for production of new variants. Mutation rates are modulated by other factors such as determinants of the template sequence and structure involved in viral replication.

RNA-dependent RNA polymerases (RdRps) are multi-domain proteins able to catalyze RNA-template dependent formation of phosphodiester bonds between ribonucleotides in the presence of divalent metal ion [7–9]. In most viruses, RNA polymerase lacks proofreading capability, with some exceptions such as *Nidovirales* order (to which the *Coronavirus* genus belongs), that stands out for having the largest RNA genomes. *Nidoviruses* are characterized by a complex machinery dedicated to RNA synthesis, that is operated by non-structural proteins (nsps), being produced as cleavage products of the ORF1a and ORF1b viral polyproteins [10] to facilitate virus replication and transcription.

The SARS-CoV-2 RdRp (also named nsp12) is a key component of the replication/transcription machinery. SARS-CoV-2 shares a high homology for nsp12 compared to SARS-CoV, suggesting that its function and mechanism of action might be well conserved [11]. This has been confirmed by a recent cryo-EM structural study obtained for SARS-CoV-2 nsp12 [12]. In SARS-CoV, an exonuclease activity with proofreading function has been reported for the nsp14 (ExoN), and a homologue nsp14 protein is found in the SARS-CoV-2 as well [11, 13]. ExoN increases the fidelity of RNA synthesis by correcting nucleotide incorporation errors made by RdRp [14]. Genetic inactivation of the coronavirus ExoN results in a 21-fold decrease in replication fidelity compared to wild type SARS-CoV [15]. Moreover, Kirchdoerfer and colleagues showed the involvement of nsp7 and nsp8 in the formation of a supercomplex with RdRp in SARS-CoV [16], and this has been confirmed also for SARS-CoV-2 in a recent study unveiling the structure of SARS-CoV-2 RdRp/nsp7/nsp8 complex [12]. This complex ensures RdRp processivity, becoming fundamental in the transcription fidelity. Nevertheless, the critical SARS-CoV RdRp residues involved in ExoN, nsp7 and nsp8 interaction have still to be identified.

RdRps are considered among primary targets for antiviral drug development, against a wide variety of viruses. Some RdRp inhibitors have been considered to target SARS-CoV-2: Favipiravir [17], Galidesivir [18], Remdesivir [19] and Ribavirin [20]. Interestingly, the docking site is not located in proximity to the catalytic domain of the RdRp [21]. In addition, other possible drugs such as Filibuvir, Cepharanthine, Simeprevir and Tegobuvir, are predicted to be potential inhibitors of RdRp [22]. Naturally occurring mutations in critical residues for drug efficacy can lead to drug resistance phenomena, with a significant loss in the binding affinity of these molecules to the RdRp.

We focused our study on SARS-CoV-2 mutations in order to assess if new viral variants were spreading across the Countries. This characterization of SARS-CoV-2 variants could lead to better therapeutics treatments, vaccines design and diagnostics approaches.

## Methods

SARS-CoV-2 virus reference sequence used for the analysis was deposited in January 2020 by Wu and coworkers [11] formerly called “Wuhan seafood market pneumonia virus” (WSM, NC_045512) (https://www.ncbi.nlm.nih.gov/nuccore/NC_045512**)**. GISAID database (https://www.gisaid.org/) filtered from December 2019 up to March 13th, 2020 was used to collect 220 SARS-CoV-2 complete genomes of different patients all around the world (i.e. China, USA, Canada, Australia, United Kingdom, Germany, France, Japan, Italy, Switzerland, Singapore, Luxembourg, Netherlands, Spain, Portugal, Sweden, Czech Republic, Thailand, India, Cambodia, Hong Kong, Finland, Singapore, and Ireland) taking into particular consideration those deposited during the development of European outbreaks. Only complete genomes (28000–30,000 bps) were analyzed.

Clustal Omega, Serial Cloner and Blast tools were used to conduct multiple sequence alignment, comparing WSM sequence to sequences isolated from patients, whereas Swiss Model and Ez-mol were used for protein modeling.

The statistical analysis was performed by R software. We first checked the normality of data distribution with the Shapiro–Wilk test, expressing the continuous variables as median and range (min–max). Categorical variables were expressed as absolute frequency and percentages. Nonparametric Mann–Whitney and Fisher-Exact tests were used to compare the number of mutations per genome with at least one of the selected mutations with respect to the group of genomes that do not present the specific mutation analyzed. All p-values were calculated from 2-sided tests using 0.05 as the significance level.

## Results

### Identification of recurrent mutation hotspots in different geographic areas

A database of 220 complete SARS-CoV-2 patient-isolated genome sequences randomly collected from the GISAID database were aligned and compared to the WSM SARS-CoV-2 reference genome. In particular, 5 patient-isolated genomes were submitted to the GISAID database in December 2019 (2.3%), 67 in January 2020 (30.45%), 67 in February 2020 (30.45%) and 81 (36.8%) up to the 13th of March 2020. About 33.6% of complete genomes belong to patients aged less than 44 years old, which is the average age of the patients included in the database. The majority of patients are men (55.5%).

**Fig. 1:**
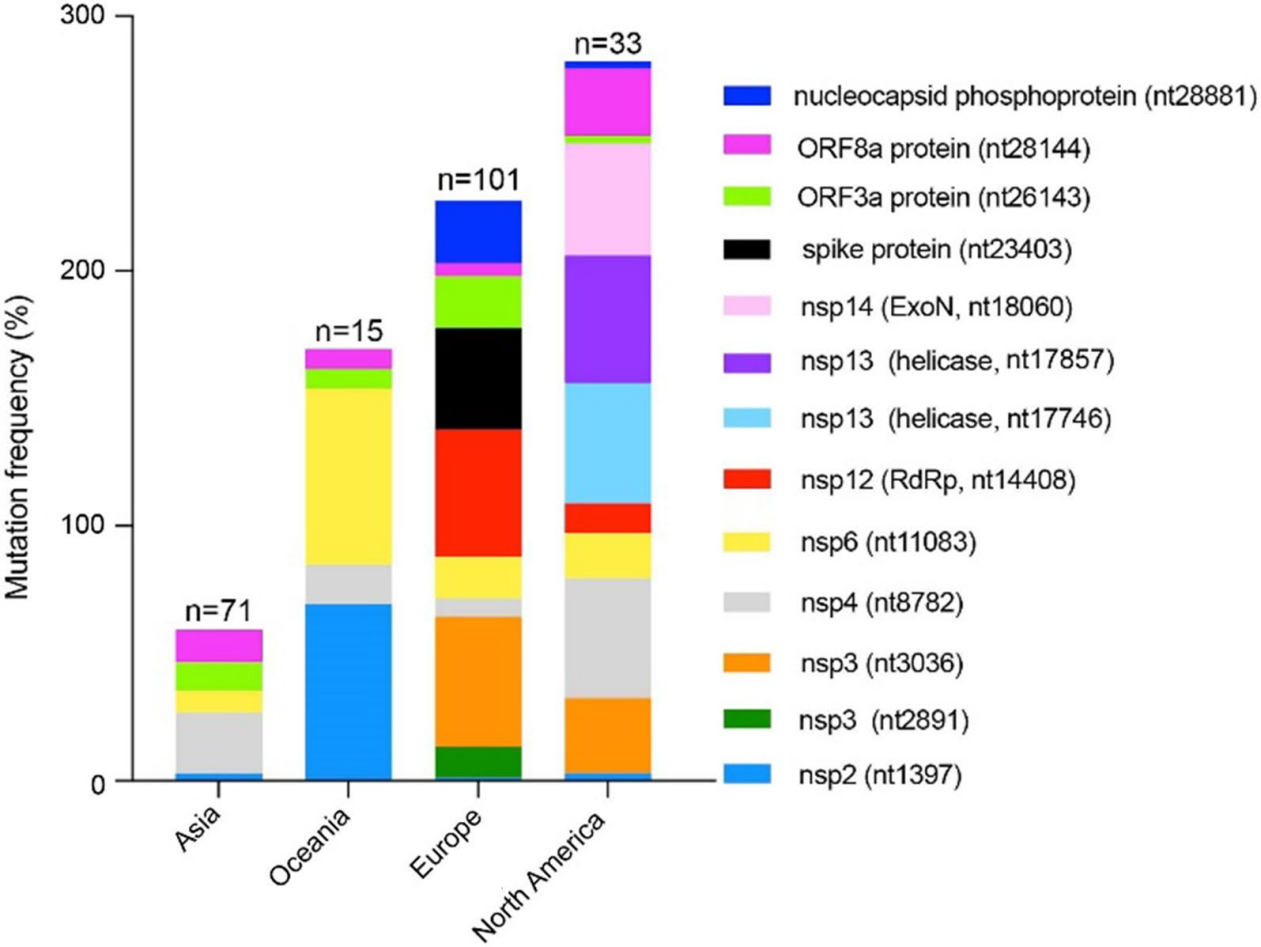
SARS-CoV-2 mutation frequency in different geographic areas. Eight novel recurrent hotspots mutations (namely 1397, 2891, 14408, 17746, 17857, 18060, 23403 and 28881) and 5 hotspots already reported in literature (namely 3036, 8782, 11083, 28144 and 26143) were subdivided into 4 geographic areas: Asia (n = 71), Oceania (n = 15), Europe (n = 101), North America (n = 33). The mutation frequency was estimated for each of them, by normalizing the number of genomes carrying a given mutation in a geographic area, by the overall number of retrieved genomes per geographic area; the graph shows the cumulative mutation frequency of all given mutations present in each geographic area. Mutation locations in viral genes are reported in the legend as well as the proteins (i.e. non-structural protein, nsp) presenting these mutations. The figure shows that genomes from European and North American patients present an increase in mutation frequency compared to Asia. It is also possible to observe that Europe and North America show a differential pattern of mutations: mutation 14408 (red), 23403 (black), 28881 (electric blue) and 26143 (light green) are present mostly in Europe, whereas 18060 (pink), 17857 (purple) and 17746 (light blue) are present mostly in North America

We divided our dataset into 4 geographic areas: Asia, Oceania, Europe, North America (Fig. 1). Within each area we performed alignment analysis comparing patients’ genomes with the reference sequence. The Asian group comprises genomes obtained from patients located in China, Japan, South-East-Asia and India. The Oceanian group comprises genomes from Australian patients, whereas the European one includes every genome obtained from patients located in each one of the European states (Spain, Portugal, United Kingdom, Netherlands, Italy, Germany, Switzerland, France, Luxemburg, Sweden, Finland, Denmark and Belgium). Finally, the North America group contains genomes from US and Canadian patients.

We evaluated the distribution of SARS-CoV-2 mutations through different geographic areas (see Fig. 1), calculating the mutation frequency within these 4 geographic areas, by normalizing the number of genomes carrying a given mutation per geographic area.

We confirmed the occurrence of mutations located at positions 3036, 8782, 11083, 28144 and 26143 [23–25, 33]. Moreover, we highlighted the presence of additional “conserved mutations” in all the geographic areas, taking into account only those occurring more than 10 times in our database. Those with a lower occurrence were not reported. These mutations were found in position 1397, 2891, 14408, 17746, 17857, 18060, 23403, 28881, belonging to ORF1ab (1397 nsp2, 2891 nsp3, 14408 RdRp, 17746 and 17857 nsp143, 18060 nsp14), S (23403, spike protein) and ORF9a (28881, nucleocapsid phosphoprotein) sequences, respectively.

We found that 3 out of the 12 most frequent mutations (positions 3036, 8782 and 18060) were silent, whereas one mutation (position 11083) was outside the ORF sequence. On the other hand, mutations 1397, 2891, 14408, 17746, 17857, 23403, 26143, 28144 and 28881 resulted in amino acid changes as follows: 1397 (V to I), 14408 (P to L), 17746 (P to L), 17857 (C to Y), 23403 (D to G), 26143 (G to V), 28144 (L to S). Mutation located at position 28881 is related to a double codon mutation, inducing the substitution of two amino acids, namely 28881 (R to K) and (G to R). The new amino acid present in 1397 (V to I), 14408 (P to L), 17746 (P to L), 17857 (C to Y), 26143 (G to V) and 28144 (L to S) had a similar isoelectric point compared to the original amino acid present in the reference protein sequences, with the exception of the mutations at positions 23403 (D to G), 28881 (R to K) and 28881 (G to R), where the mutated amino acid has a significantly different isoelectric point. Further studies are needed to determine whether these mutations have an impact on proteins’ function and structure. We noted that the number and the occurrence of each mutation increase in genomes found out of Asia, reaching a maximum in genomes found in Europe and North America. We also noted that the viral strains found in Europe and North America are derived from the L-“strain” originated in Asia [23].

### Characterization of geographically distinct hotspots over time

In order to determine the appearance of each mutation, we analyzed each genome from each geographic area over time, by classifying them according to the timing of sample collection, as indicated in the GISAID database. According to this analysis, 6 time subgroups were defined, namely December 2019 (genomes from 5 patients), 1st–15th Jan. 2020 (genomes from 15 patients), 16th–31st Jan. 2020 (genomes from 52 patients), 1st–15th Feb. 2020 (genomes from 13 patients), 16th–29th Feb 2020 (genomes from 55 patients) and 1st–13th Mar 2020 (genomes from 80 patients).

**Fig. 2:**
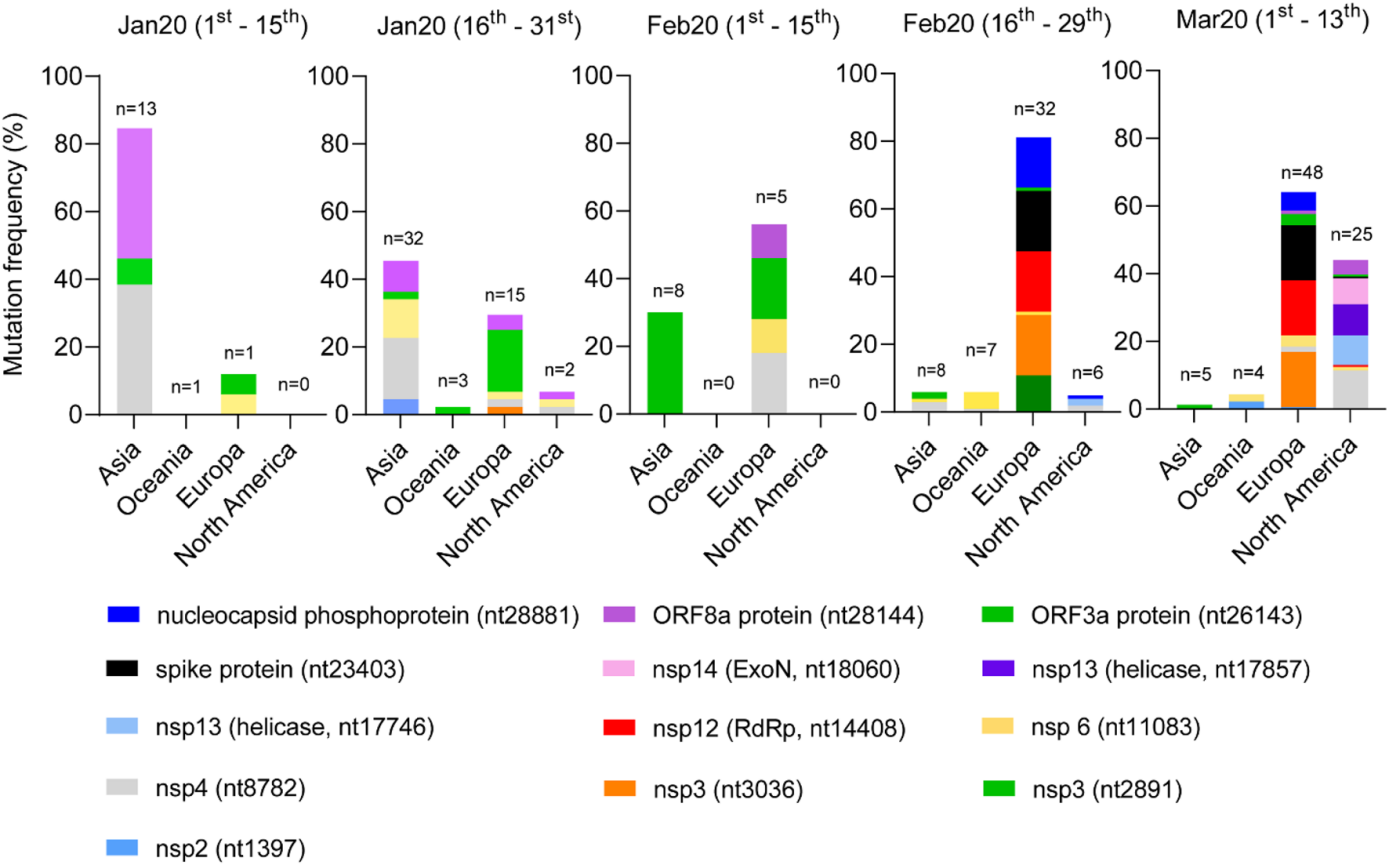
SARS-CoV-2 Mutation occurrence over time divided per geographic area. Eight novel recurrent hotspots mutations (namely 1397, 2891, 14408, 17746, 17857, 18060, 23403 and 28881) and 5 hotspots already reported in literature (namely 3036, 8782, 11083, 28144 and 26143) were subdivided first into 5 period subgroups: December 2019 (n = 5), 1st–15th Jan. 2020 (n = 15), 16th–31st Jan 2020 (n = 52), 1st–15th Feb 2020 (n = 13), 16th–29th Feb 2020 (n = 55) and 1st–13th Mar 2020 (n = 80). Next, for each time group, a further subclassification per geographic area (Asia, Oceania, Europe and North America) was performed (number of genomes in each area are reported in the figure inset). The number of mutations in each area was normalized by the number of genomes analyzed for each period of time. This figure shows that mutation frequency increases over time during viral spread out of Asia. No mutations were observed in the Asian genomes analyzed in December 2019. In the time group of February 16th–29th, a defined cluster of mutations emerged in Europe; in March 1st–13th, a different cluster of mutations emerged in North America

The number of mutations (normalized by the population taken into account for each period of time) increases over time during viral spread out of Asia (see Fig. 2). No mutations were observed in the Asian genomes analyzed in December 2019. Interestingly, a different pattern of mutations was observed in Europe between January and February, when a new mutation, at position 14408, emerged (depicted in red). This mutation is located in the RdRp gene. Also starting from February 2020, the emergence of additional new mutations (i.e. 23403, 28881 and 2891–black, electric blue, dark green, respectively) is observed. Over time, we also noted an increase in the frequency of mutation 3036 (orange), already present in mid-January (2.2%).

Moreover, a different pattern of hotspot mutations is clearly distinguishable in viral genomes detected in North American patients starting from March 2020, when the outbreak of positive cases was reported in the US and Canada. In this group, three novel mutations (17746, 17857 and 18060–light blue, purple and light pink, respectively) were reported. Interestingly, viral genomes present in North American patients carrying RdRp mutation (14%) do not carry any of the European specific mutations.

### Mutations hotspots pattern after February 9th, 2020

**Fig. 3:**
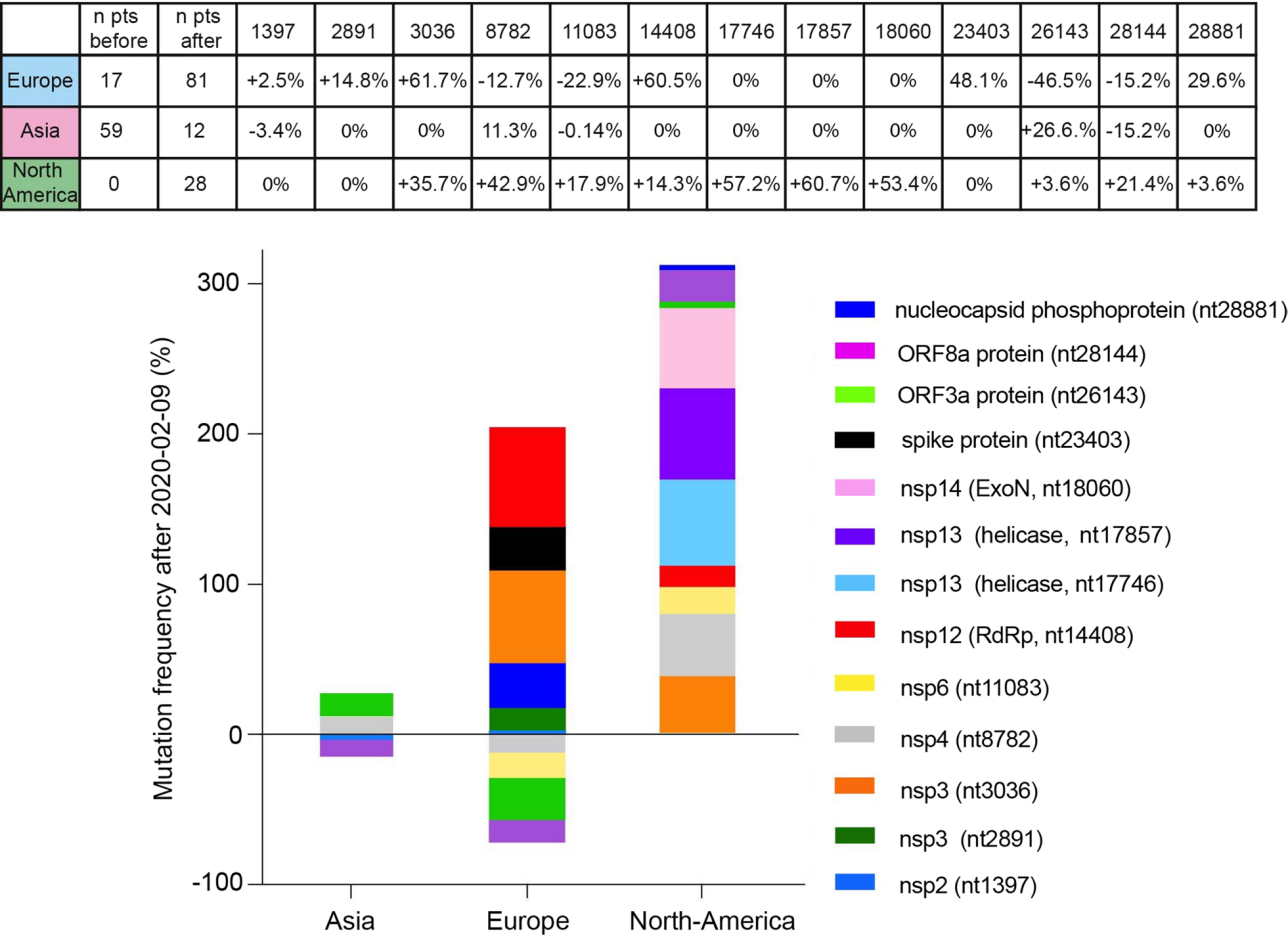
Increment of SARS-CoV-2 mutation frequency after RdRp mutation appearance per geographic area. The increment of mutation frequency before and after February 9th, 2020 across the different geographic areas (Asia, Europe, North America) is shown. The figure shows a diminishment of Asian mutations (i.e. 1397, 8782, 11083, 26143 and 28144) that is simultaneous with the appearance of new mutations such as 2891, 3036, 23403 and 28881, when RdRp novel European mutation located at 14408 (in red) occurred. The upper table shows the increment or decrement for each single mutation, per geographic area

Given the importance of RdRp for viability and replication of RNA viruses, mutations in this gene are statistically less likely to occur. However, in some cases, such as in poliovirus, episodes of drug-resistance induced by a point mutation in RdRp have been reported [26]. In our database, the first appearance of a silent RdRp mutation (nt 14804) is manifested on February 9th, 2020 in UK (England), while a different RdRp mutation (nt 14408, amino acid P to L) is observed for the first time in Italy (Lombardy) on February 20th, 2020, when a dramatic increase of the number of European infected patients was reported from the WHO website [27]. We evaluated the increase/decrease of each mutation frequency before and after February 9th, 2020 across the different geographic areas (Fig. 3). In particular, we observed a strong increase (+60.5%) of genomes carrying the 14408 mutation (affecting RdRp) in Europe, together with an increase of genomes carrying the 3036 mutation (+61.7%), the 23403 mutation (48.1%) and the 28881 mutation (+29.6%) (see upper table Fig. 3).

### Simultaneous occurrence of RdRp mutation with other mutations

Next, we analyzed genomes collected after February 9th 2020, when mutation in RdRp gene was reported in the database for the first time. For the purpose of analysis, we divided the genomes into two groups: group 1 contains genomes with mutation in position 14408 (RdRp) (n = 53, 4 North America and 49 European), and group 2 without RdRp mutation (n = 84).

Genomes in group 1 showed an increased number of mutations compared to group 2. In particular, group 1 shows 6 genomes with two mutations (11.3%), 25 genomes with three mutations (47.2%), 21 genomes with four mutations (39.6%), and 1 genome with 5 mutations (1.9%). In group 1, the most reported mutations are the ones in positions 3036, 14408, 23403 and 28881. Regarding genomes in group 2, 20 do not carry any mutations (23.8%), 25 genomes have a single mutation (29.8%), 19 genomes have two mutations (22.6%), 6 genomes have three mutations (7.1%), 9 genomes have four mutations (10.7%), 2 and 3 genomes have five and six mutations respectively (2.4% and 3.6%). In group 2, the most reported mutations are located at positions 8782, 11083, 17746 and 17857.

**Fig. 4:**
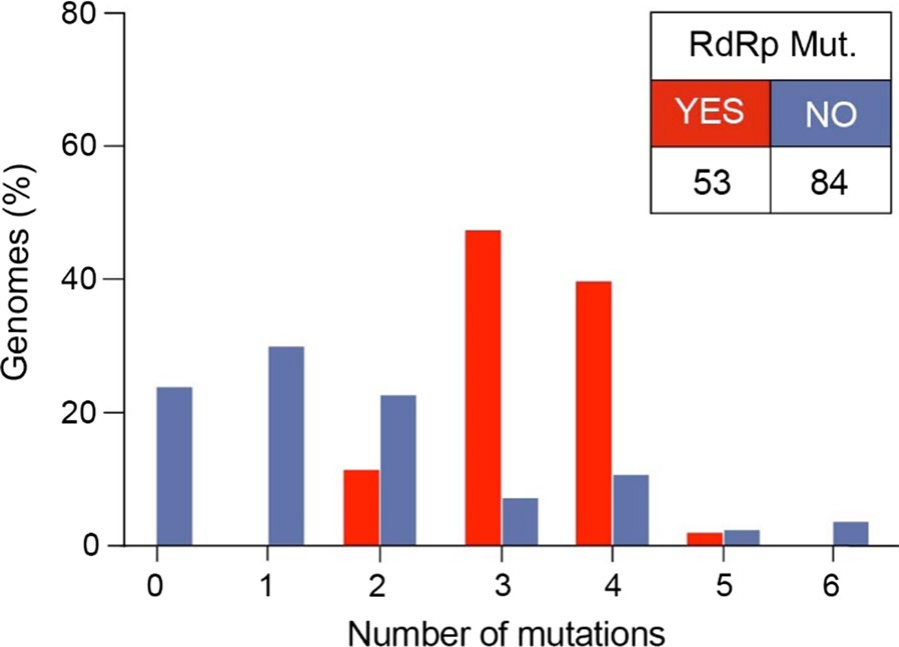
Number of SARS-CoV-2 mutations associated with the RdRp mutation. Genomes were subdivided into two groups: group 1 contains genomes with mutation in position 14408 (RdRp) (n = 53, 4 North America and 49 European), and group 2 without RdRp mutation (n = 84). We further subdivided group 1 and 2 by the number of mutations present in the genome. Genomes in group 1 (red bars) showed an increased number of mutations compared to group 2 (grey bars). Most genomes of groups 1 (86.8%) have at least 3 or 4 mutations, whereas 76.2% of genomes of group 2 have less than 2 mutations. We found that viral strains with RdRp mutation have a median of 3 point mutations [range: 2–5], whereas viral strains with no RdRp mutation have a median of 1 mutation [range: 0–3] (p value < 0.001, Mann–Whitney test)

The distribution between the two groups in terms of number of mutations is statistically relevant (Fisher-Exact test, *p* value < 0.001). In particular, group 1 and 2 are significantly different in terms of the distribution of genomes having 0, 1, 3 and 4 numbers of mutations (Fisher-Exact test, p < 0.001) (Fig. 4). This difference, instead, is insignificant when the number of mutations is 2, 5 or 6.

We found that viral strains with RdRp mutation have a median of 3 point mutations [range: 2–5], whereas viral strains with no RdRp mutation have a median of 1 mutation [range: 0–3] (p value < 0.001, Mann–Whitney test). The different distribution between the two groups relative to the number of mutations is statistically significant (Fig. 4).

We also analyzed the most frequent mutations detected: the ones in positions 3036, 23403 and 28881 (in Europe), and the ones in positions 17746, 17857 and 18060 (in North America). Viral genomes carrying each one of these mutations were compared with viral genomes without mutations, by using Mann–Whitney test for paired-groups comparison analysis. Genomes carrying mutations in positions 3036, 23403, 28881, 17746, 17857 and 18060 show a median of 3–4 mutations (range [2:5]), whereas genomes carrying none of them have a median of 1 or 2 mutations (range [0:3], p-value < 0.001, Mann–Whitney test). This difference is statistically significant and implies that if one of those mutations is present, other mutations are more likely to occur.

### Homology study of mutant RdRp protein

Among all mutation sites analyzed, RdRp mutant is particularly interesting given that the enzyme is directly involved in viral replication and its fidelity determines the mutagenic capabilities of SARS-CoV-2. Due to the high homology between RdRps of SARS-CoV and SARS-CoV-2, we aligned SARS-CoV-2 RdRp reference sequence with the reported catalytic site sequence of SARS-CoV RdRp.

The amino acid substitution 323 (P to L) (due to nucleotide mutation 14408) falls outside the catalytic site, in a region that in SARS-CoV is reported to be an Interface Domain, a still poorly characterized surface structure, supposedly implicated in the interaction with other proteins which may regulate the activity of RdRp [16]. To this regard, it is well-known that SARS-CoV RdRp forms a hollow cylinder-like supercomplex with nsp7 and nsp8, which confer processivity to RdRp [28]. Additionally, replication supercomplex interacts with nsp14, an exonuclease having the Nidovirales-typical proofreading capability. This activity is important in the context of the mutation rate and for controlling the fidelity in RNA replication. However, critical RdRp residues involved in this interaction are still to be identified, and for this reason further studies are needed to assess the possible role of mutation 14408 concerning RdRp fidelity.

## Discussion

In the present work we have compared the SARS-CoV-2 reference genome to those exported from the GISAID database with the aim of gaining important insights into virus mutations, their occurrence over time and within different geographic areas.

We observed that after February 2020, when the first locally transmitted SARS-CoV-2 cases out of Asia were reported, viral genomes presented different point mutations, clearly distinguishable within different geographic areas. Over time, we were able to identify three recurrent mutations in Europe (in positions 3036, 14408 and 23403) and 3 other different mutations in North America (in positions 17746, 17857 and 18060). So far, these mutations have not been detected in Asia. The number and the occurrence, as well as the median value of virus point mutations registered out of Asia, increase over time.

In our study, we found that the RdRp mutation, located at position 14408, which is present in European viral genomes starting from February 20th, 2020, is associated with a higher number of point mutations compared to viral genomes from Asia. Given that RdRp works in a complex machinery that includes proofreading activities (in cooperation with other viral cofactors, like ExoN, nsp7 and nsp8), it is tempting to speculate that this mutation has contributed in impairing its proofreading capability. One possible mechanism could involve a minor change in the RdRp structure, without affecting its catalytic activity, that might nonetheless alter its binding capability with other cofactors such as ExoN, nsp7 or nsp8, thus altering the mutation rate. This could explain the increased number of mutations that we observed in Europe since February 2020. Further studies are needed to determine whether the observed cluster mutations originate from the same molecular mechanism. Further studies are also needed to determine whether the mutation in RdRp results in increased viral replication.

Some polymerase inhibitors [29, 30] are currently being tested in clinical studies to target SARS-CoV-2 RdRp, including Favipiravir [17, 19], Galidesivir [18], Remdesivir [19], Ribavirin [20], Penciclovir [31], Galidesivir [32] and Ponatinib [33]. Additionally, other drugs such as Simeprevir (FDA approved HCV protease inhibitor), as well as Filibuvir and Tegobuvir (both RdRp inhibitors) [22], are predicted to bind RdRp by molecular docking studies. In particular, a putative docking site was identified in a hydrophobic cleft very close to the mutated site 323 (P to L), corresponding to mutation 14408 identified in our study [22]. Naturally occurring mutations in RdRp can potentially lead to drug-resistance phenomena, as already observed previously [19, 34, 35]. Alternatively, it might induce a significant decrease in drug-RdRp complex binding affinity. This could lead to different effectiveness of antiviral treatments where mutation 14408 is present. For this reason, due to the high frequency of RdRp mutation in the infected population, it is important to characterize the impact of 14408 mutation on the activity of RdRp and its susceptibility to antiviral drugs.

## Conclusions

We identified novel mutation hotspots in the SARS-CoV-2 genome sequences. Interestingly, some appeared after February 2020, only in European patients. Among these hotspots, one mutation in position 14408 is located within the RdRp protein and is associated with an overall increased mutation rate. An in silico analysis comparing annotated functional domains of SARS-CoV and SARS-CoV-2 sequences, showed that this particular mutation occurs in the so-called RdRp interface domain, a still poorly characterized surface structure, involved in protein–protein interactions [16]. The role for the RdRp interface domain requires further investigations, and in particular the effect of mutation in position 14408, its interaction with other cofactors (such as ExoN, nsp7 and nsp8), possibly affecting its proofreading activity and potentially altering its mutation rate. It is also essential to understand if the described mutations could result in the emergence of drug-resistance viral phenotypes. Our data may help the development of diagnostic and therapeutic strategies and the study of potential drug resistance mechanisms.

## Data Availability

The datasets analysed during the current study are available in the GISAID (https://www.gisaid.org/) and GenBank (https://www.ncbi.nlm.nih.gov/nuccore/NC_045512) repositories.

## Abbreviations

RdRp: RNA-dependent RNA polymerase
nsp: Non-structural protein
SARS-CoV: Severe acute respiratory syndrome coronavirus
SARS-CoV-2: Severe acute respiratory syndrome coronavirus 2 (COVID-19)
MERS-CoV: Middle east respiratory syndrome coronavirus
ExoN: Exonuclease
WHO: World Health Organization
WSM: Wuhan Seafood Market
